# ADXS11-001 immunotherapy targeting HPV-E7: final results from a Phase II study in Indian women with recurrent cervical cancer

**DOI:** 10.1186/2051-1426-2-S3-P92

**Published:** 2014-11-06

**Authors:** Robert G Petit, Ajay Mehta, Minish Jain, Sudeep Gupta, Rajnish Nagarkar, Vijay Kumar, Sumana Premkumar, Rakesh Neve, Subhashini John, Partha Basu

**Affiliations:** 1 Advaxis. Inc, NJ, USA; 2 Central India Cancer Research Institute, Nagpur, Maharashtra, India; 3 Ruby Hall Clinic, Maharashtra, India; 4 Tata Memorial Hospital, Maharashtra, India; 5 Curie Manavata Cancer Center, Maharashtra, India; 6 MV Hospital and Research Center, Chennai, Tamil Nadu, India; 7 Dr. Kamakshi Memorial Hospital, Chennai, Tamil Nadu, India; 8 Smt. Kashibai Navale Medical College and General Hospital, Pune, Maharashtra, India; 9 Christian Medical College Vellore, Vellore, Tamil Nadu India; 10 Chittaranjan National Cancer Institute, Kolkata, West Bengal, India

## Background

ADXS11-001 immunotherapy is a live attenuated *Listeria monocytogenes *(*Lm*) bioengineered to secrete a tLLO-HPV-16-E7 fusion protein targeting HPV-transformed cells. The *Lm *vector serves as its own adjuvant and infects APC where it cross presents HPV-E7-tLLO fusion peptide, stimulating MHC class 1 and 2 pathways resulting in HPV-E7 specific T cell immunity. *Lm*-LLO-E7-015 (Clinical Trials Registry India #CTRI/2010/091/001232), is a randomized Phase II study designed to evaluate the safety and efficacy of ADXS11-001 +/- Cisplatin in patients with recurrent cervical cancer in India.

## Methods

110 patients were randomized to either 3 doses of ADXS11-001 at 1 × 10^9 ^cfu or 4 doses of ADXS11-001 at 1 × 10^9 ^cfu with Cisplatin chemotherapy (40 mg/m^2^, weekly x5). Naproxen and oral Promethazine were given as premedications. Patients received CT scans at baseline and 3, 6, 9, 12 and 18 months. The primary endpoint was overall survival.

## Results

Final 12-month survival was 32% (35/109), 18-month survival was 22% (24/109) and 24-month survival was 18% (16/91). The response rate was 11% (5 CRs and 6 PRs/109) with tumor responses observed in both treatment arms; 31 additional patients had stable disease **≥**3 months, for a disease control rate of 38% (42/109). Average duration of response in both treatment groups was 9.5 months. Long term survivors (LTS; ≥18 months) included patients with tumor shrinkage and patients with increased tumor burden as their best tumor response overall; 8% (2/24) of LTS failed at least two prior treatments; and 25% (3/11) of LTS were ECOG performance status 2 at baseline. Activity against different high-risk HPV strains was observed. The addition of Cisplatin to ADXS11-001 did not improve survival or tumor response over ADXS11-001 alone but contributed to toxcity. Baseline ECOG performance status, type of prior therapy, or aggressiveness of disease had no significant effect on survival or tumor response. ADXS11-001 was well tolerated as 62% (68/109) of patients reported no adverse events and 38% (41/109) of patients reported only mild transient adverse events (G1-2) that occurred on the day of infusion. The incidence of SAEs possibly related/ related to ADXS11-001 was 1% G3 (0% G4-5).

## Conclusions

ADXS11-001 appears to have significant clinical activity in patients with recurrent cervical cancer. The observed prolonged survival, objective tumor responses, and stabilization of recurrent disease compare favorably with more toxic chemotherapy treatment options and support ADXS11-001 as an active agent in the treatment of cervical cancer.

